# Supplementary material for: WordCommentsAnalyzer: A windows software tool for qualitative research
Source: F1000Res. 2018 Sep 4;7:536. Originally published 2018 May 3. [Version 2] doi: 10.12688/f1000research.14819.2 (PMC6137411; doi:10.12688/f1000research.14819.2)
Supplement: Supplementary file 3 [file f1000research-7-17614-s0002.tgz › b2a79496-4ada-47c4-9682-a99ce867836d.zip!/Eight Iranian Health Journals/Archives of Rehabilitation_65.docx]

© Archives of Rehabilitation. This work is licensed under CC BY-NC 4.0 (https://creativecommons.org/licenses/by-nc/4.0/).

Volume & Issue: دوره 4 - شماره 1

Title: بررسی موارد آتاکسی حاد مراجعه کننده به بخش اعصاب بیمارستان مفید از مهر ماه ۸۰ تا مهر ماه ۸۲

http://rehabilitationj.uswr.ac.ir/article-1-588-fa.html

هدف: اصطلاح آتاکسی زمانی عنوان می گردد که اختلال در کنترل وضعیت و حرکت بوجودآید. این کنترل توسط مخچه و سیتم های دریافت کننده آن از لب فرونتال به ستون خلفی نخاع صورت می گیرد. تظاهر اولیه معمولا اختلال راه رفتن به عدم کنترل در راه رفتن است که بصورت با فاصله راه رفتن و تلو تلو خوردن بروز می نماید.آتاکسی حاد به آتاکسی گفته می شود که بطور ناگهانی اتفاق افتاده باشد. با توجه به آنکه آتاکسی حاد یکی از علل شایع مراجعه به مراکز اعصاب کودکان می باشد و تا بحال بررسی در این مورد در ایران صورت نگرفته است بر آن شدیم تا این تحقیق را انجام دهیم. روش بررسی: از مهر 80 تا مهر 82 تمام کودکانی که با شکایت اختلال تعادل در راه رفتن بطور ناگهانی، به بخش اعصاب کودکان بیمارستان مفید مراجعه نمودند را مورد معاینه قرار داده و از بین آنها 100 مورد آتاکسی حاد تشخیص و مورد مطالعه قرار گرفت. در مورد بیماران بستری و آزمایشات و تصویربرداری لازم به عمل آمد. سپس نتایج بدست آمده در پرسشنامه ای جمع آوری و اطلاعات استخراج گردید. یافته‌ها: در نتایج بدست آمده شایعترین علت، آتاکسی حاد مخچه ای مشاهده شد که در تمامی این کودکان با یک عفونت تب دار قلی همراه بوده است. در اکثریت موارد عفونت قبلی آبله مرغان گزارش گردید، یک مورد مننگوانسفالیت اریونی و یک مورد آنسفالیت هرپسی نیز مشاهده شد. پس از آن علت مسمومیت دارویی با بیشترین طیف سنی کودکان (2-4سال) قرار داشت. نتیجه‌گیری:‌ سایر علل به ترتیب علل پلی نوروپاتی عفونی، میگرن، اپسوکلونوس مایوکلونوس، تومورمغزی، A.D.E.M ،MS و اپی لپسی هستند.

Title: بررسی رابطه عملکرد برتری دستی با مهارت‌های خودمراقبتی و مقایسه آن بین دانش‌آموزان عقب‌مانده ذهنی و عادی

http://rehabilitationj.uswr.ac.ir/article-1-589-fa.html

هدف:‌ برتری دستی و مهارت های خود مراقبتی در کاردرمانی جایگاه ویژه ای دارد و از مهارت های بنیادی در توان بخشی می باشد. این تحقیق توصیفی- تحلیلی در مدارس آموزش و پرورش منطقه 11شهر تهران در سال 1381بر روی 61دانش آموز عادی (32عدد راست دست، 29عدد چپ دست) و 59 دانش آموزش عقب مانده ذهنی آموزش پذیر (40عدد راست دست، 19عدد چپ دست) پایه های تحصیلی آمادگی، اول و دوم انجام گرفت. روش بررسی:‌ میانگین سنی دانش آموزان عادی کم تر از دانش آموزان عقب مانده ذهنی بود (P=0/000) برخی از داده ها از طریق پرسش نامه های ویژه دادهای اختصاصی از طریق اجرای آزمون اختصاصی به صورت عملکردی از آزمودنی به عمل آمد. برتری دستی و مهارت های خود مراقبتی آنها مورد مقایسه و ارتباط آن بین دو گروه محاسبه شد. یافته‌ها: بین میانگین قدرت گریپ دست راست و چپ آزمودنی های راست دست مدارس عادی اختلاف معناداری وجود داشت (P=0/011) اما در آزمودنی های چپ دست اختلاف معناداری وجود نداشت (P=0/234). میان مهارت های خود مراقبتی و قدرت گریپ آزمودنی های عقب مانده ی ذهنی و عادی ارتباط معناداری وجود دار (P=0/000) مقایسه مهارت دستی دو گروه از نظر آماری اختلاف معناداری را نشان می دهد (P=0/000) نتیجه‌گیری: مقایسه هم بستگی بین مهارت دستی با مهارت خو مراقبتی در آزمودنی های عقب مانده ی ذهنی معنادارتراز آزمودنی های عادی بود(P<0/05) و هم بستگی بین برتری دستی و یا بیش تر از برتری دستی با چشم بود. در مقایسه ی دانش آموزان راست دست و چپ دست مدارس عادی بر اساس رتبه ی تولد اختلاف معناداری وجود دارد (P=0/963) پیشنهاد می شود برای ارزیابی و تعیی برتری دستی از چند جنبه (قدرت ، مهارت و از طریق پرسش نامه) مورد توجه  قرار گیرد و به صرف نوشتن با دست اکتفاء نگردد.

Title: بررسی میزان شیوع افسردگی در پناهندگان افغانی مقیم اردوگاه بردسیر کرمان

http://rehabilitationj.uswr.ac.ir/article-1-591-fa.html

هدف:‌ نیاز به مهاجرت معمولا تابع روابط پیچیده عوامل اقتصادی، اجتماعی، خانوادگی و سیاسی است. از مهم ترین عوامل موثر در مهاجرت را می توان عدم دستیابی به تحصیلات، کار، خدمات و احترام به حقوق اولیه ی انسانی نام برد. این تحقیق با توجه به تعدد بالای مهاجران افغانی در ایران و با هدف بررسی میزان شیوع افسردگی در پناهندگان افغانی مقیم اردوگاه بردسیر کرمان صورت گرفته است. روش بررسی:‌ در این بررسی 300 نفر (162زن و 138مرد) از افغانیان ساکن در این اردوگاه انتخاب و پرسش نامه ی استاندارد شده ی افسردگی بک و پرسش نامه دموگرافیک در مورد آن ها پرشد. نحوه نمونه گیری به صورت (خوشه ای – خوشه ای ) و مطالعه به صورت مقطعی انجام گرفت. یافته‌ها: در نتایج به دست آمده شیوع کلی افسردگی در این مهاجرت 53 که بیشترین شدت افسردگی در گروه سنی 20-29 سال بود(البته رابطه بین سن و افسردگی معنی دار نبود) در این مطالعه بین شیوع افسردگی و جنس رابطه معنی دار وجود داشت P<0/04 یعنی شیوع افسردگی در زن ها 57/4، در مردان 47/8 بود.هرچند میزان افسردگی در افراد مجرد بیش تراز افراد متاهل بود اما این رابطه معنی دار نبود و بیش ترین شدت افسردگی در افرادی با طول پناهندگی (141-150) ماه دیده شد. نتیجه‌گیری: به طور کلی شیوع افسردگی در پناهندگان به جز جنس ،ارتباط چندانی با عوامل دموگرافیک نداشت و به طور عمده عواملی پس از مهاجرت بر شیوع افسردگی تاثیر گذار بودند.

Title: بررسی و مقایسه میانگین نمره آزمون استریوگنوز دهانی در کودکان مبتلا به شکاف کام و کودکان سالم

http://rehabilitationj.uswr.ac.ir/article-1-594-fa.html

هدف: از آنجا که حواس دهانی نقش بسزایی در رشد و اصلاح گفتار ایفا می کند. این پژوهش بر آن است که حواس دهانی را در افراد مبتلابه شکاف کام 5-9ساله بررسی و با کودکان طبیعی هم سن مقایسه کند. در صورتی که بین میانگین نمرات آزمون های استریوگنوز دهانی دو گروه فوق تفاوت معنی داری وجود داشته باشد ، نتیجه می گیریم که در درمان اختلالات گفتاری کودکان شکاف کامی می توان از روش های تقویت کننده حواس دهانی نیز بهره جست. روش بررسی: روش این پژوهش تحلیلی – مقطعی است که 66کودک سالم و 30کودک مبتلابه شکاف کام توسط آزمون های تشخیص شکل و طرح از نظر استریگنوز دهانی مورد بررسی و مقایسه قرار گرفتند. همچنین تاثیر متغیرهای سن و جنس، وجود شکاف کام، نوع شکاف کام و تعداد عمل جراحی بر روی میزان استریوگنوز دهانی بررسی شد. یافته ها: -1 در دو گروه کودکان سالم و کودکان مبتلا به شکاف کام، میزان ماستریوگنوز دهانی تفاوت معنی داری نشان می دهد. -2 در دو گروه کودکان سالم و کودکان مبتلا به شکاف 5-9ساله، با افزایش سن ، میزان استریوگنوز دهانی پیشرفت می کند. ساله ، با افزایش سن ، میزان استریوگنوز دهانی پیشرفت می کند. -3 جنسیت بر روی استریوگنوز دهانی تاثیر ندارد. -4در کودکان مبتلا به شکاف کام 5-9ساله بین نمرات تشخیص شیء و نوع شکاف کام رابطه ای وجود ندارد ، اما این رابطه بین نمره تشخیص طرح و نوع شکاف کام برقرار است. توجیه تفاوت بین این دو نتیجه دشوار می باشد. نتیجه‌گیری: در کودکان مبتلا به شکاف کام ، با افزایش تعداد عمل جراحی میزان استریوگنوز دهانی کاهش می یاید.

Title: بررسی شیوع اختلال رفتاری در دانش آموزان مقطع ابتدایی شهر ابهر

http://rehabilitationj.uswr.ac.ir/article-1-595-fa.html

هدف: پژوهش حاضر به منظور بررسی میزان شیوع اختلال رفتاری در دانش آموزان دختر و پسر مقطع ابتدایی مدارس دولتی شهر ابهر در سال تحصیلی 1380-1381 انجام گرفته است در این پژوهش هم چنین رابطه ای بین میزان اختلال رفتاری با عامل های جنسیت، پیشرفت تحصیل، میزان تحصیل و وضعیت شغلی والدین مورد بررسی قرار گرفت. روش بررسی: پرسش نامه ی اختلال رفتاری راتر ویژه ی معلمان که رفتارهای کودکان را در کلاس درس مورد بررسی قرار می دهد بر روی 600دانش آموز (300دختر و 300پسر) که با استفاده از روش نمونه برداری چند مرحله ای انتخاب شدند ، اجرا شد. یافته‌ها: یافته های پژوهش نشان داد که، میزان شیوع اختلال رفتاری بر اساس نقطه برش پیشنهادی 9، در دانش آموزان %43/3 است، میزان شیوع اختلال رفتاری در دختران %42 و در پسران 44/7 است. نتایج نشان  دادند که اختلالات رفتاری در %6/3 کل آزمودنی ها شدید است. این میزان در دختران برابر %5/6 و در پسران برابر با %7 است. نتیجه‌گیری:‌ بین میزان اختلال رفتاری دختران و پسران تفاوت معنی دار وجود نداشت اما بین اختلال رفتاری و پیشرفت تحصیلی هم بستگی منف معنی داری وجود داشت، هم چنین بین میزان تحصیل مادر در اختلال رفتاری دانش آموزان رابطه معنی دار وجود داشت اما میزان تحصیل پدر تاثیر معنی داری نداشت. بین میزان اختلالات رفتاری دانش آموزان و وضعیت شغلی پدر رابطه معنی دار وجود داشت که نتایج به دست آمده مورد بحث و تفسیر قرار گرفته است.

Title: تأثیر سیتالوپرام، آلپرازولام و کلومیپرامین در کاهش علائم منفی بیمار مبتلا به اسکیزوفرنیا

http://rehabilitationj.uswr.ac.ir/article-1-598-fa.html

هدف: علائم منفی به منزله یکی ار علائم پنج گانه جدول تشخیصی اسکیزوفرنیا در DSMIV، از موانع مهم بر سر راه توان بخشی این دسته از بیماران و ایجاد ارتباط از سوی آنان با سایرین می باشد و از جمله روش های مورد بحث برای تقلیل این علائم استفاده از داروهایکمکی به انضمام درمان های جاری است. در این پژوهش نقش سیتالوپرام ، کلومیپرامین و آلپرازولام را در کاهش علائم منفی بررسی می نماییم. روش بررسی: چهل بیمار اسکیزوفرنیک بستری در بلوک سالمندان مرد در مرکز روان پزشکی رازی به طور راندوم انتخاب و به طور دوسوکور به صورت چهار گروه به ترتیب تحت درمان با سیتالوپرام 20میلی گرم ، آلپرازولام ./75 میلی گرم ، کلومپرامین 25میلی گرم و بالاخره پلاسبو به اضافه رژیم درمانی جاری شان ( شامل آنتی سایکوتیک های تیپیکال) قرار گرفتند. پس از دو هفته دوز داروها به دو برابر فزایش یافت و این روند به مدت دو هفته دیگر ادامه یافت و سپس داروها قطع گردید. وجود و شدت علائم منفی در ابتدا و قبل از آغاز درمان و سپس در انتهای هفته دوم و بالاخره در انتهای هفته چهارم ا استفاده از مقیاس SANSکنترل و ثبت گردید. در طی این مدت (4هفته) هیچ گونه عارضه جانبی خاصی که موجب نارحتی بیماران یا قطع دارو گرددد مشاهدهنشد. پس از استخراج اطلاعات به دست آمده و با استفاده از روش محاستبات آماری zو chisquare(x2-test) ارتباط بین متغیرها سنجیده گردید. یافته ها: سه داروی سیتالوپرام ، آلپرازولام و کلومیپرامین موجب تقلیل شدت علائم منفی به ترتیب در 80/،50/،50/بیماران گروه خود گردیدند. در اکثریت قریب به ابفلاق بیماران ، این کاهش محدود به 20/از سطح پایه باقی ماند. در این بین کلومیپرامین در دو مورد به میزان 40علائم منفی را کاهش داد هم چنین کلومیپرامین بیش از دو تای دیگر بر علائم شدید منفی تاثیر داشت اما تاثیر داروها به طور کلی بر روی بیماران دارای علائم منفی متوسط و خفیف بیش از علائم منفی شدید بود. تقلیل علائم منفی به طور غیر وابسته به یکدیگر صورت گرفته بود و در بیماران مختلف از ترکیب متنوعی برخوردار بود. بین گروه سالمندان و غیر سالمندان از بابت تاثیر داروها تفاوتی ملاحظه نگردید. هیچ یک از بیماران گروه کنترل تحت تاثیر پلاسبو با تقلیل علائم منفی مواجه نگردیدند. نتیجه گیری: سه داروی سیتالوپرام ، آلپرازولام و کلومیپرامین می توانند در کاهش علائم منفی در بیماران مبتلا به اسکیزوفرنیا نقش مفیدی را ایفا نمایند. در این بین تاثیر مثبت سیتالوپرام با توجه به احتمال عوارض تداخل دارویی کم تر نسبت به دوتای دیگر قابل توجه است.

Title: گزارش یک مورد نادر سندرم لوب فرونتال

http://rehabilitationj.uswr.ac.ir/article-1-599-fa.html

هدف سندرم لوب فرونتال، اختلال دائم شخصیتی با نمای بالینی خاص است که ناشی از آسیب لوب های فرونتال است. در این سندرم ناپایداری خلقی، حملات تکراری خشونت، قضاوت اجتماعی مختل، آپاتی، بی تفاوتی، یا شک و افکار پارانوئیدی دیده می شود. در حال حاضر طبق طبقه بندی DSM-IV به عنوان یک اختلال شخصیتی ناشی از حالت طبی خاص در محور/طبقه‌بندی می‌شود. آقای 46ساله ای است که 10سال پیش به دنبال ضربه مغزی دچار بی تفاوتی، بی انگیزگی و آپاتی به صورت گوشه گیری، کم حرفی، زندگی در پارک و خیاان شده است. تابلوی بالینی در طی 10سال گذشته ثابت بوده است. بر اساس شرح حال اخذ شده از خانواده، معاینه روانی و عصبی بیمار، تصویربرداری از مغز، بررسی اعمال شناختی و آزمون های سرندشناسی و نروپسیکولوژیک عمده با هدف ارزیابی عملکرد لوب فرونتال بوده است. با توجه به شایع نبودن این اختلال و این که به ندرت در تشخیص های نهایی روان پزشک منعکس می گردد، شباهت زیادی علایم آن با سایر بیماری های روانی و تاکید بر این نکته که کلید تشخیص در این اختلال، تغییر شخصیت از سطح قبلی است و نه نوع علایم و تطابق این تغییر با یافته های تصویربرداری مغزی، بیمار فوق معرفی گردیده است.
